# Supplementary material for: Antimicrobial Properties of New Polyamines Conjugated with Oxygen-Containing Aromatic Functional Groups
Source: Molecules. 2023 Nov 20;28(22):7678. doi: 10.3390/molecules28227678 (PMC10675077; doi:10.3390/molecules28227678)
Supplement: Supplementary file 1 [file molecules-28-07678-s001.zip › molecules-2683303-supplementary.pdf]

Electronic Supplementary Information

Antimicrobial properties of new polyamines conjugated with oxygen containing aromatic functional groups

Figure S1. ^{1}H NMR spectra of the new compounds 2

Figure S2. ^{13}C NMR spectra of the new compounds........ 5

Figure S3. ATR-IR spectra of all the new compunds. 7

Figure S4. Scheme and X-ray crystallographic structure of **3a**·3HCl. 10

Table S1. Crystal data, data collection parameters, and results of the analysis. 11

Figure S5. Molar fraction species distribution diagrams. 13

Figure S6. Determination of the minimal microbicidal concentration. 15

Figure S7. Cytotoxicity determination by FACS. 16

Figure S1. ^{1}H NMR spectra of the new compounds

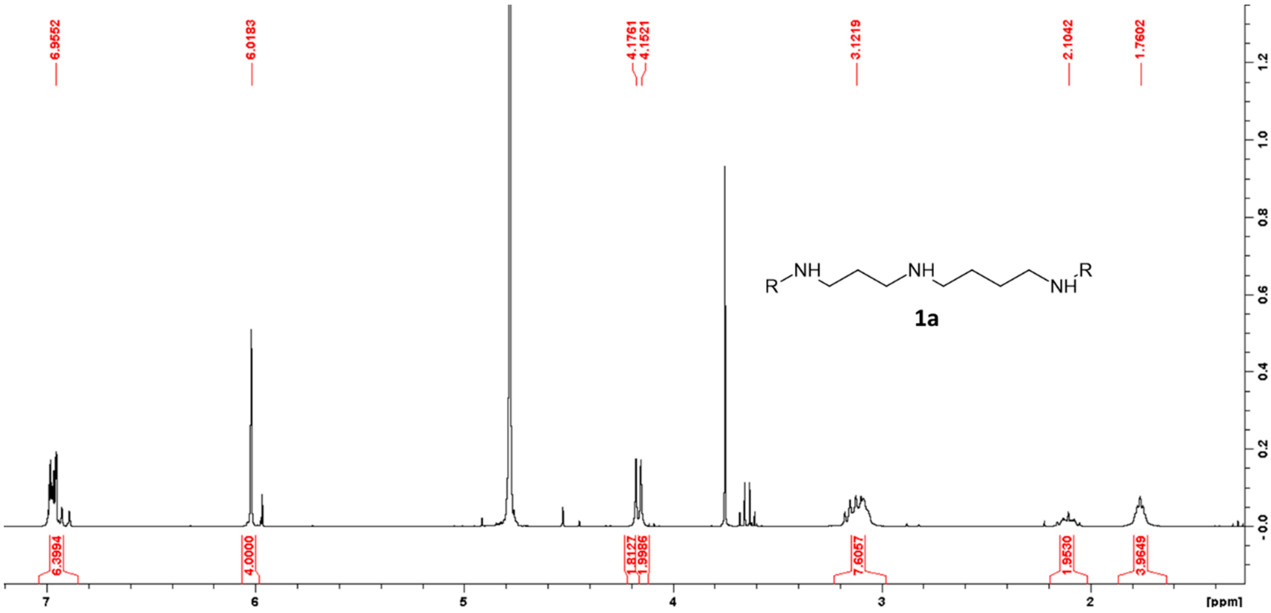

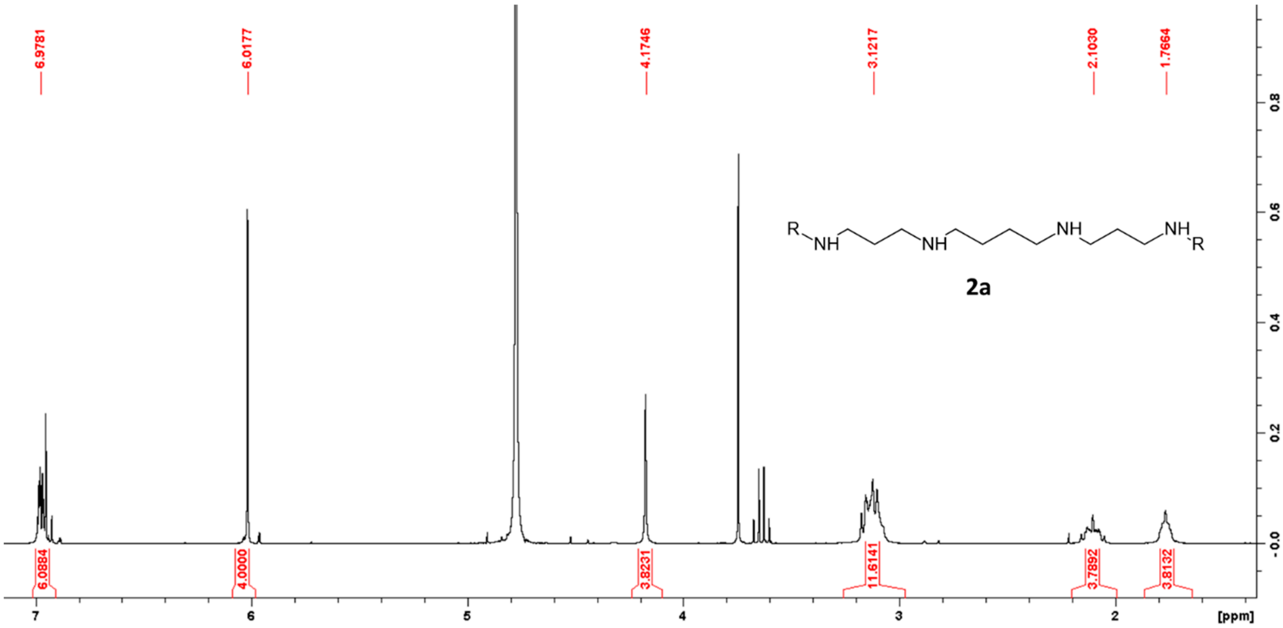

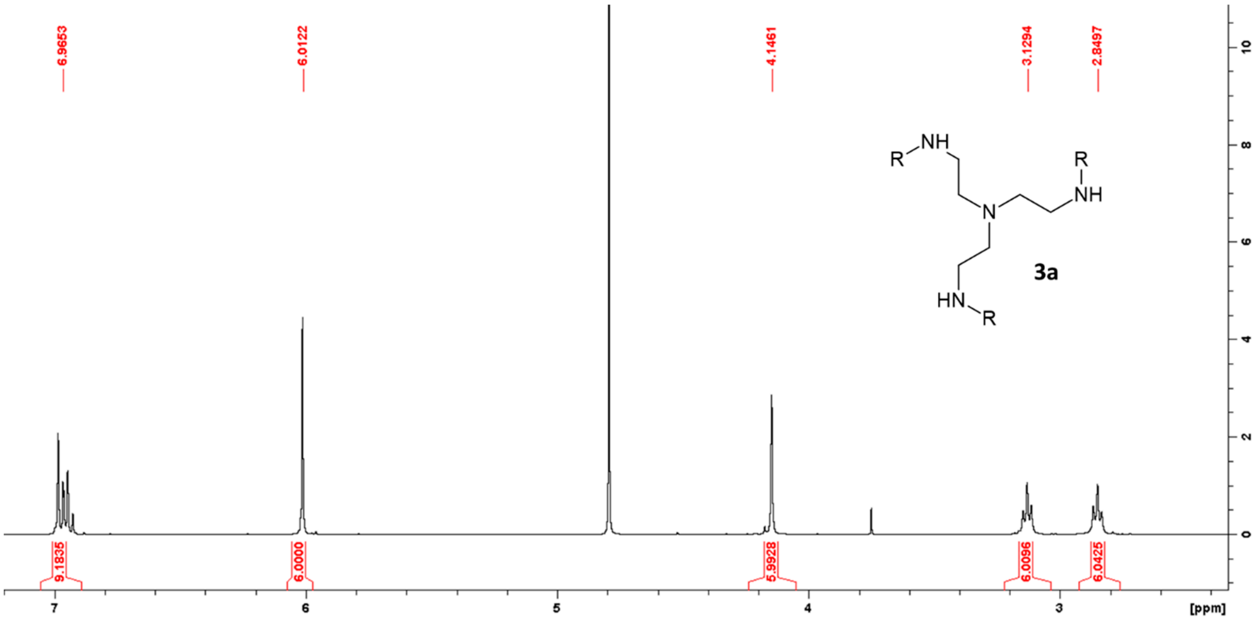
6.9653
6.0122
4.1461
3.1294
2.8497
R
NH
N
NH
HN
3a
9.1835
6.0000
5.9928
6.0096
6.0425
7
6
5
4
3
[ppm]
10
8
6
4
2
0

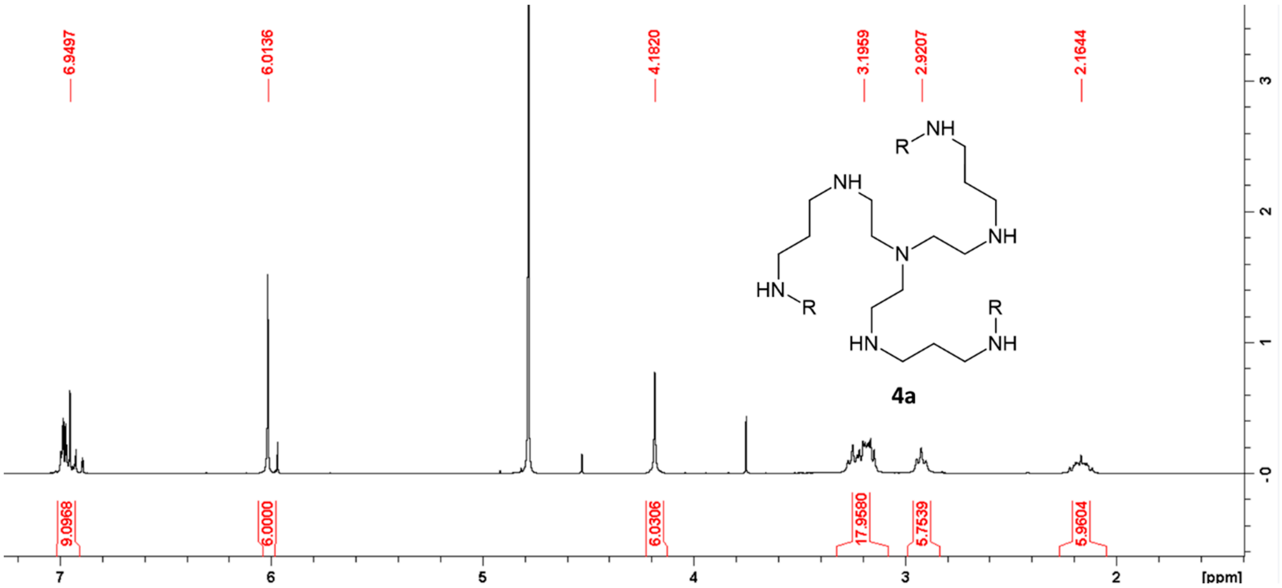
6.9497
6.0136
4.1820
3.1959
2.9207
2.1644
R
NH
N
HN
4a
9.0968
6.0000
6.0306
17.9580
5.7539
5.9604
7
6
5
4
3
2
[ppm]
3
2
1
0

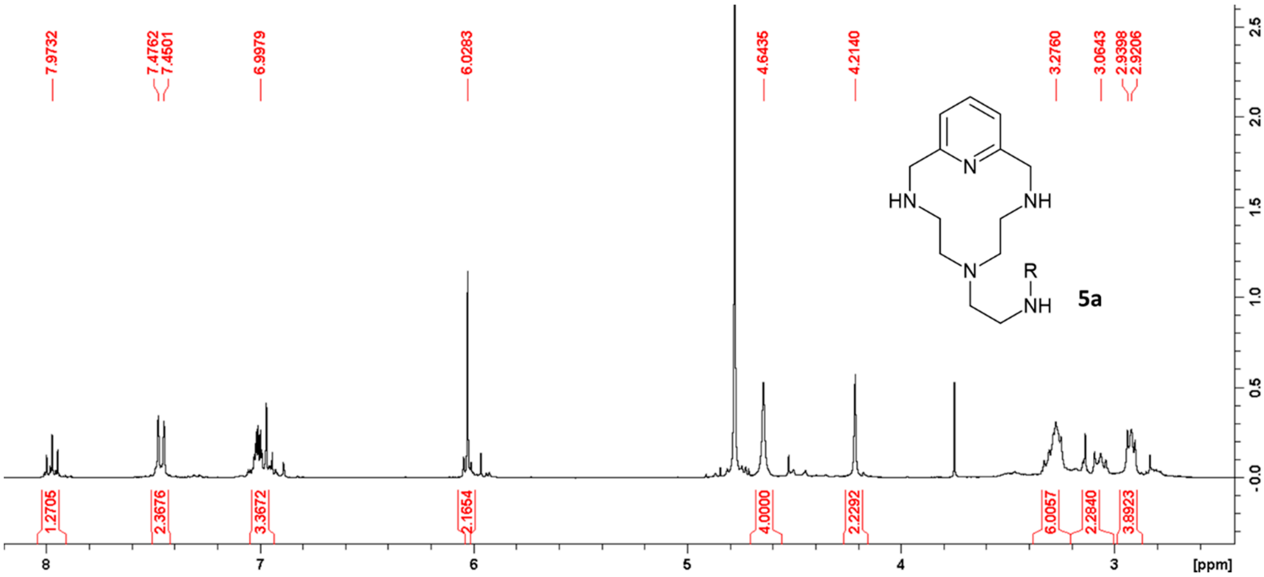
7.9732
7.4762
7.4501
6.9979
6.0283
4.6435
4.2140
3.2760
3.0643
2.9398
2.9206
1.2705
2.3676
3.3672
2.1654
4.0000
2.2292
6.0057
2.2840
3.8923
HN
NH
N
N
R
NH
5a
[ppm]

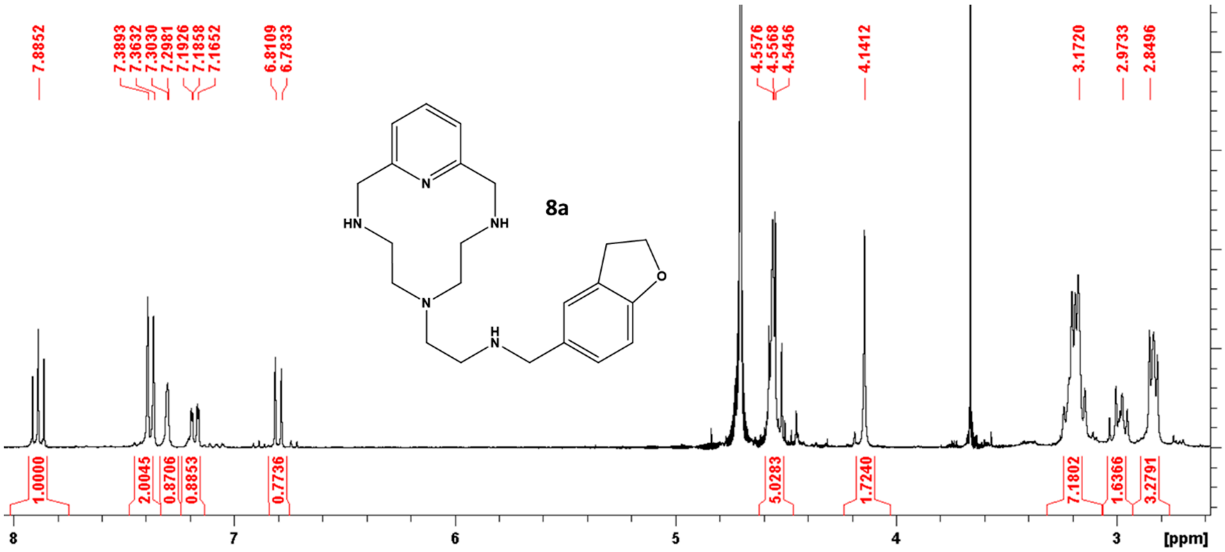
7.8852
7.3893
7.3632
7.3030
7.2981
7.1926
7.1858
7.1652
6.8109
6.7833
4.5576
4.5568
4.5456
4.1412
3.1720
2.9733
2.8496
HN
NH
N
N
H
O
8a
1.0000
2.0045
0.8706
0.8853
0.7736
5.0283
1.7240
7.1802
1.6366
3.2791
[ppm]

Figure S2. ^{13}C NMR spectra of the new compounds

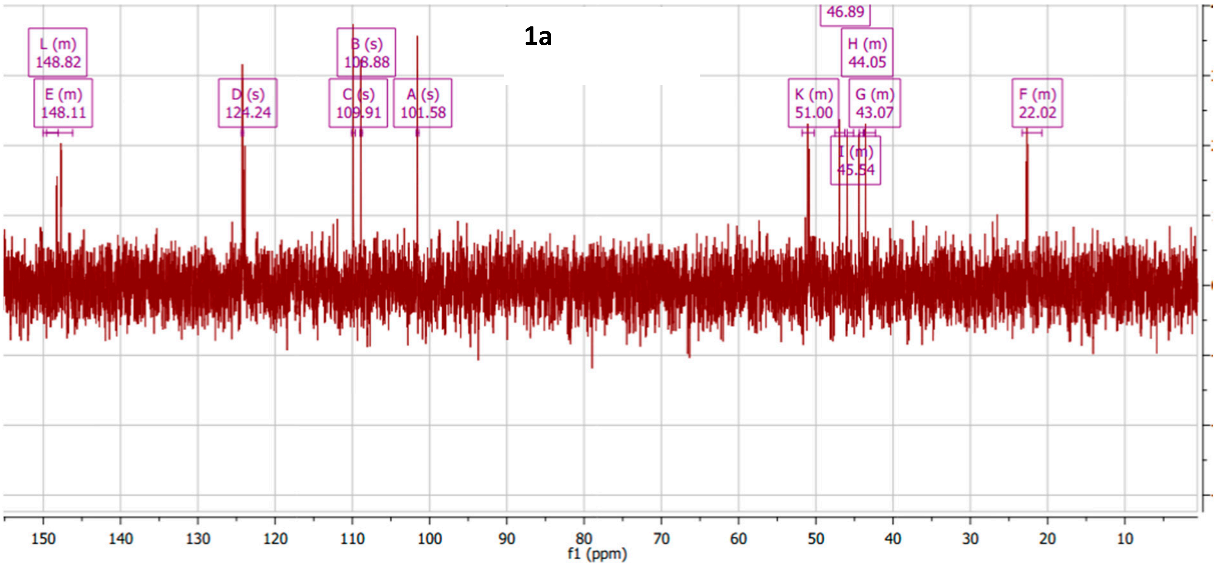

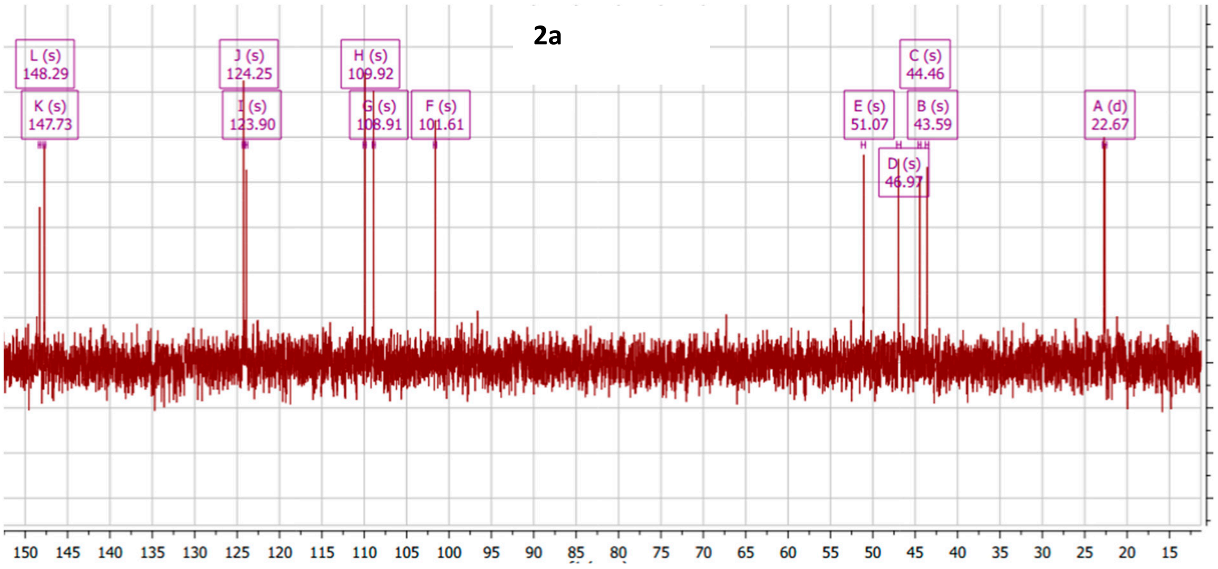

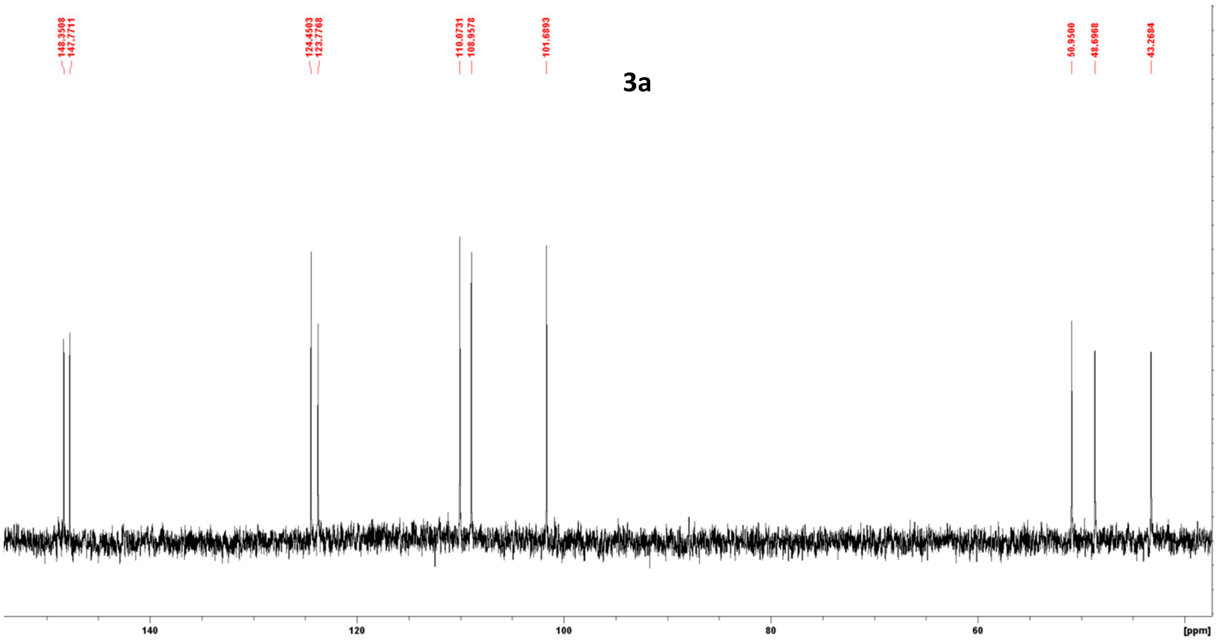
148.3508
147.7711
124.4503
123.7768
110.0731
108.9578
101.6893
3a
50.9500
48.6968
43.2684
140
120
100
80
60
[ppm]

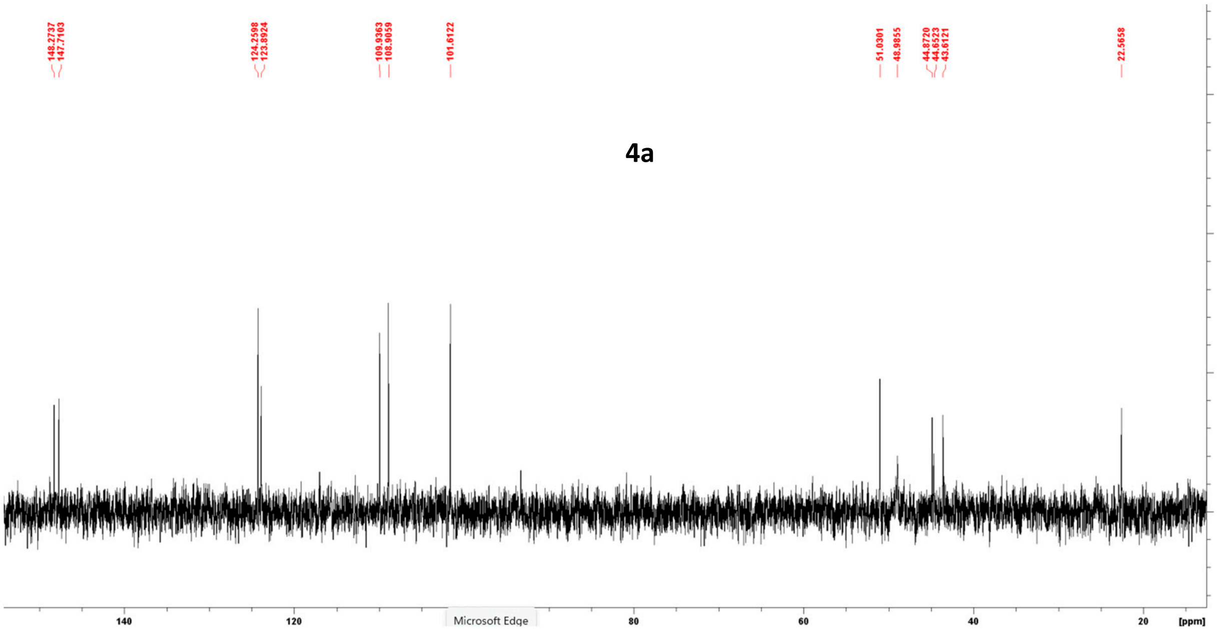
148.2737
147.7103
124.2598
123.8924
109.9363
108.9059
101.6122
51.0301
48.9855
44.8720
44.6523
43.6121
22.5658
4a
140
120
80
60
40
20
[ppm]
Microsoft Edge

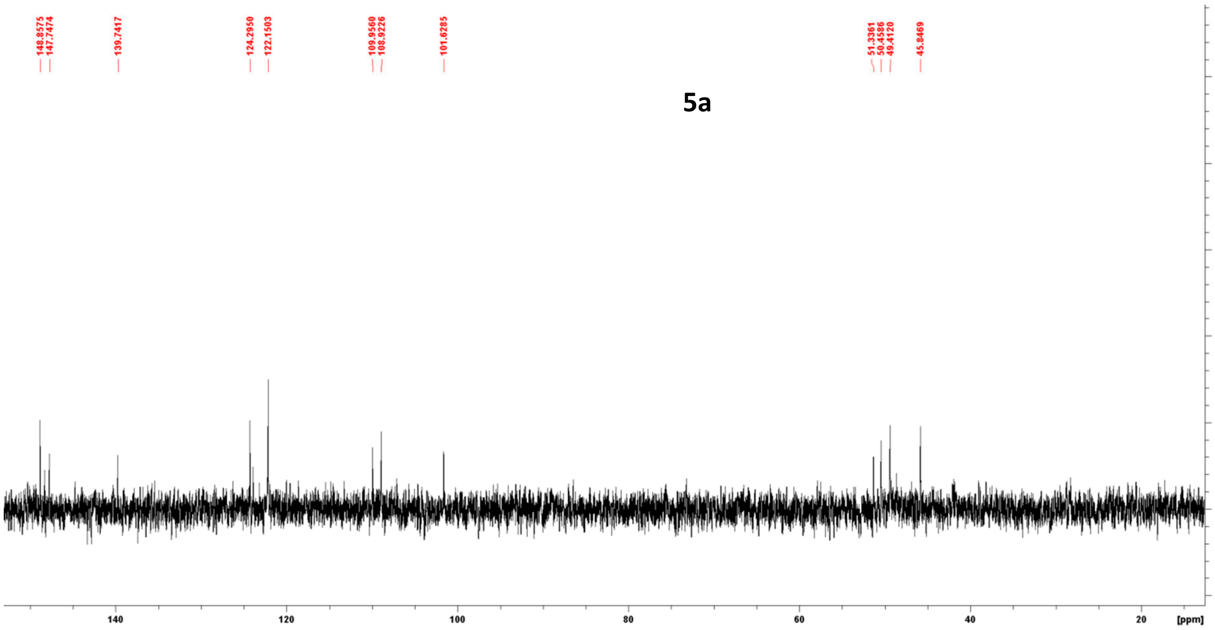
148.8575
147.7474
139.7417
124.2950
122.1503
109.9560
108.9226
101.6285
51.3361
50.4586
49.4120
45.8469
5a
140
120
100
80
60
40
20
[ppm]

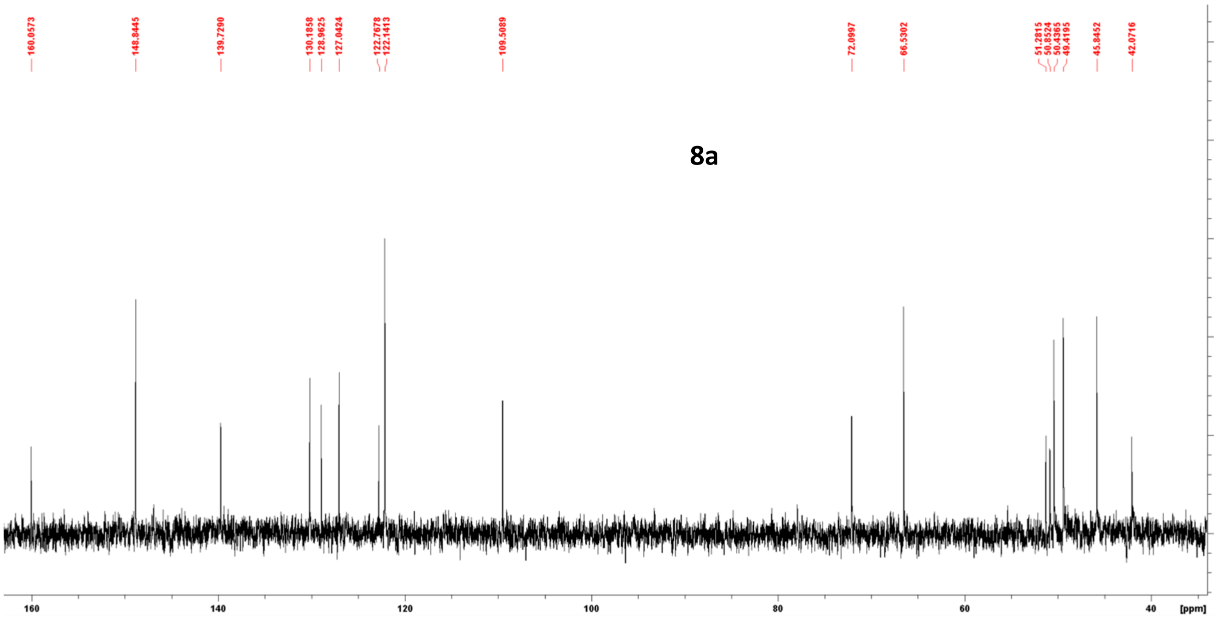
160.0573
148.8445
139.7290
130.1858
128.9625
127.0424
122.7678
122.1413
109.5089
72.0997
66.5302
51.2815
50.8524
50.4365
49.4195
45.8452
42.0716
8a
160
140
120
100
80
60
40
[ppm]

Figure S3. ATR-IR spectra of all the new compunds.

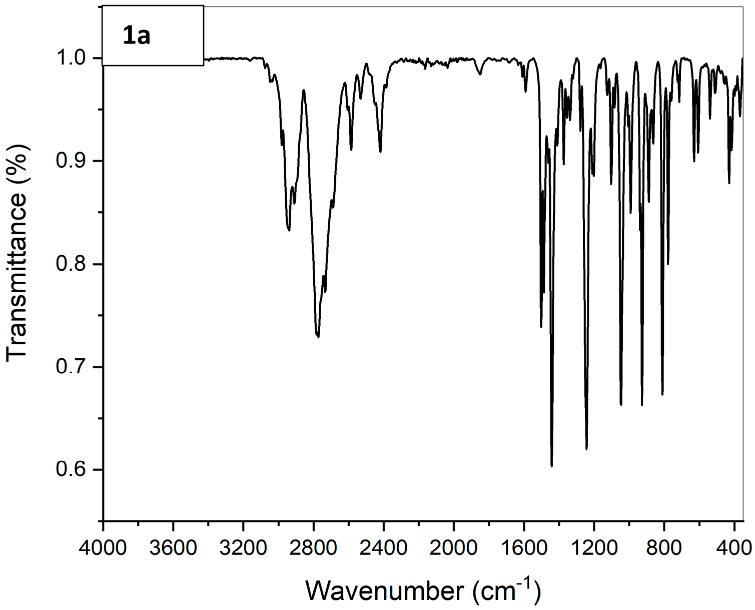

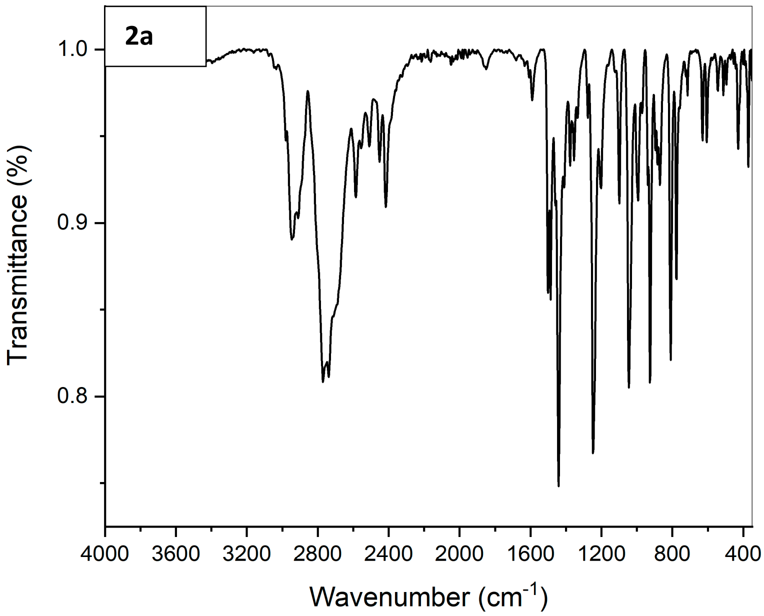

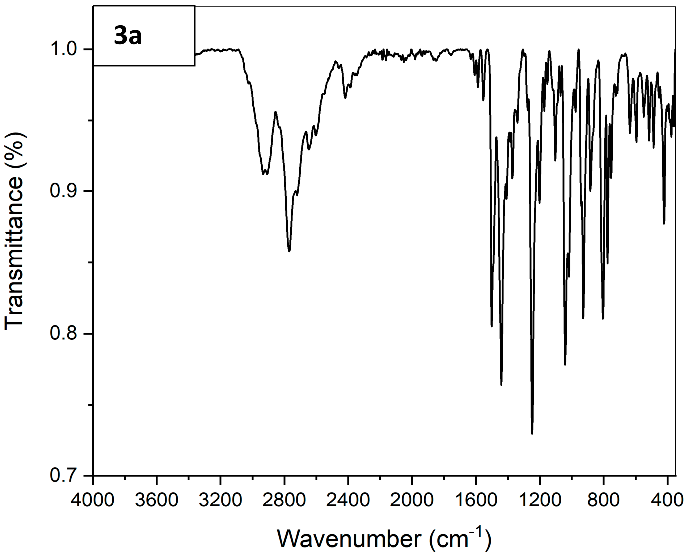
3a
Transmittance (%)
1.0
0.9
0.8
0.7
4000
3600
3200
2800
2400
2000
1600
1200
800
400
Wavenumber (cm-1)

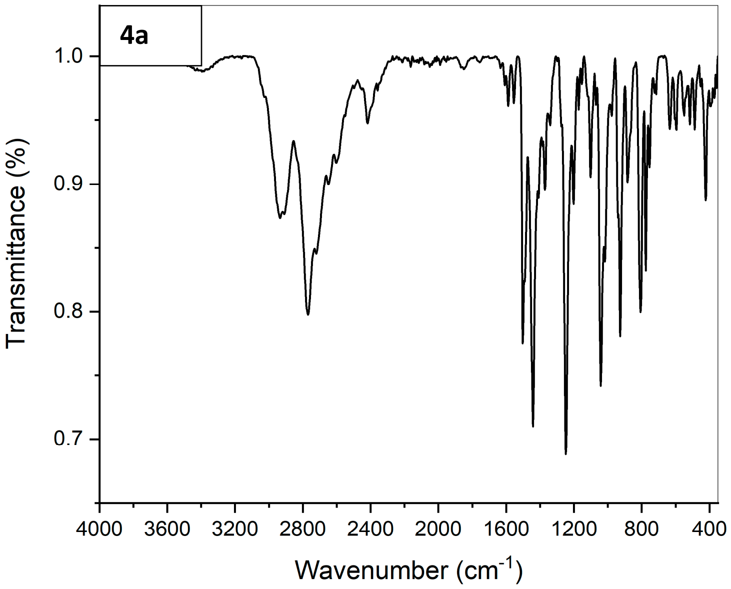
4a
Transmittance (%)
1.0
0.9
0.8
0.7
4000
3600
3200
2800
2400
2000
1600
1200
800
400
Wavenumber (cm-1)

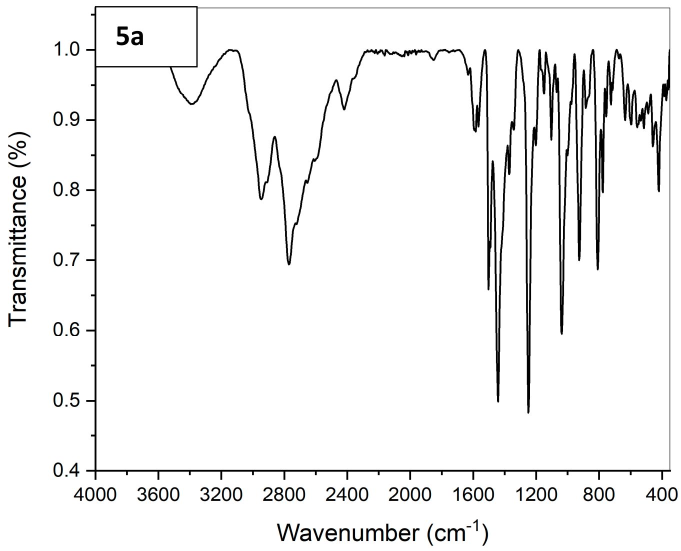
5a
Transmittance (%)
1.0
0.9
0.8
0.7
0.6
0.5
0.4
4000 3600 3200 2800 2400 2000 1600 1200 800 400
Wavenumber (cm-1)

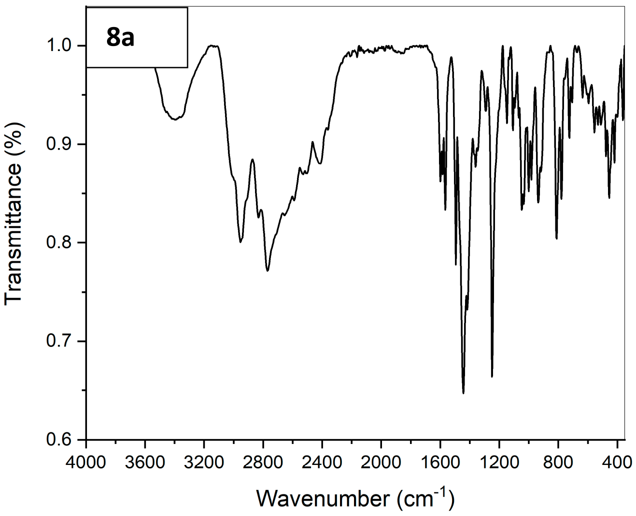
8a
Transmittance (%)
1.0
0.9
0.8
0.7
0.6
4000 3600 3200 2800 2400 2000 1600 1200 800 400
Wavenumber (cm-1)

Figure S4. Scheme and X-ray crystallographic structure of **3a**·3HCl.

Slow evaporation of vessels containing a concentrated aqueous solution of **3a**·3HCl yielded white crystals suitable for X-ray diffraction. Analysis of single crystals of the ligand was carried out with an Enraf-Nonius KAPPA CCD single-crystal diffractometer (λ = 0.71073 Å). CIFS of crystal structures have been deposited with the number 2210255.

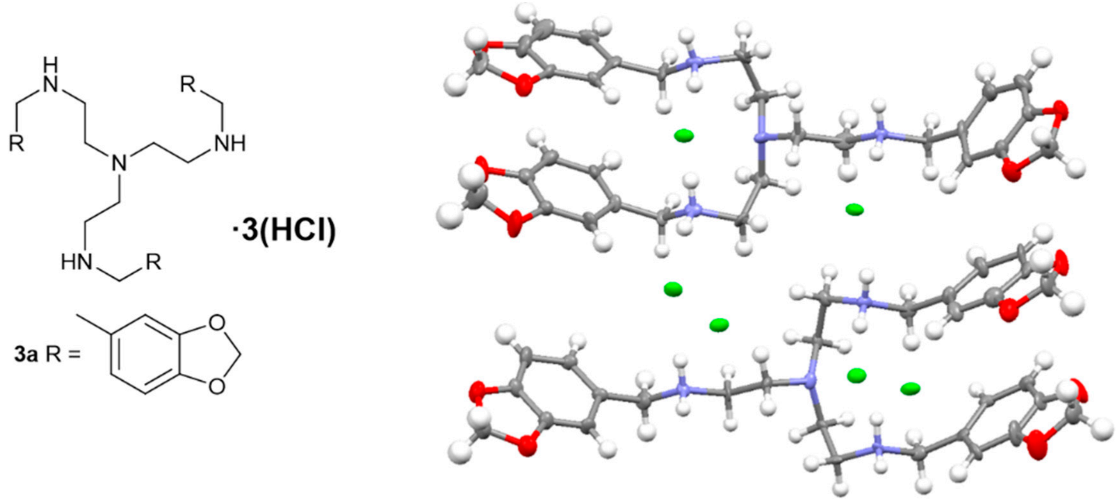

Table S1. Crystal data, data collection parameters, and results of the analysis.

Crystallographic data

Formula	$C_{30}H_{39}Cl_3N_4O_6$
Formula weight	658.00
Crystal system	triclinic
Space group	P 1
Cell<br>*a* / Å<br>*b* / Å<br>*c* / Å<br>α / °<br>β / °<br>γ / °<br>*V* / $Å^3$	 7.241(4)<br>11.317(7)<br>20.092(13)<br>77.11(3)<br>87.00(3)<br>76.795(16)<br>1562.5(16)
Z	2
T / K	150
size / mm	0.382 × 0.262 × 0.052
F_{000}	692
density / $g.cm^{-3}$	1.399
R(int)	0.1946
θ_{max} / deg.<br>θ_{min} / deg.	29.982<br>2.350
reflections<br>- collected<br>- unique	 58315<br>8581
*R*1<br>- all<br>- $F^2 > 2\sigma_{F2}$	 0.1221<br>0.0856
*wR*2<br>- all<br>- $F^2 > 2\sigma_{F2}$	 0.2312<br>0.2007
GoF	1.034
- parameters<br>- constraints<br>- restraints	777<br>0<br>39

Hydrogen bonds

D--H..A	d(D-A) / Å	d(H..A) / Å	d(D, A) / Å	ang(D,H,A) / °
N2--H2A..Cl3	0.91	2.14	3.033(14)	167
N2--H2B..Cl4[*x*+1, *y*, *z*]	0.91	2.22	3.114(12)	167
N3--H3A..Cl1[*x*+1, *y*, *z*]	0.91	2.15	3.034(10)	163
N3--H3B..Cl2	0.91	2.41	3.189(11)	144
N4--H4A..Cl2	0.91	2.25	3.145(12)	169
N4--H4B..Cl5	0.91	2.22	3.112(11)	166
N6--H6A..Cl1[*x*, *y*-1, *z*]	0.91	2.17	3.077(9)	171
N6--H6B..Cl5	0.91	2.24	3.128(10)	165
N7--H7A..Cl6	0.91	2.29	3.200(10)	173
N7--H7B..Cl4[*x*, *y*-1, *z*]	0.91	2.21	3.106(11)	166
N8--H8A..Cl3	0.91	2.20	3.071(12)	159
N8--H8B..Cl6	0.91	2.40	3.151(12)	140

Figure S5. Molar fraction species distribution diagrams.

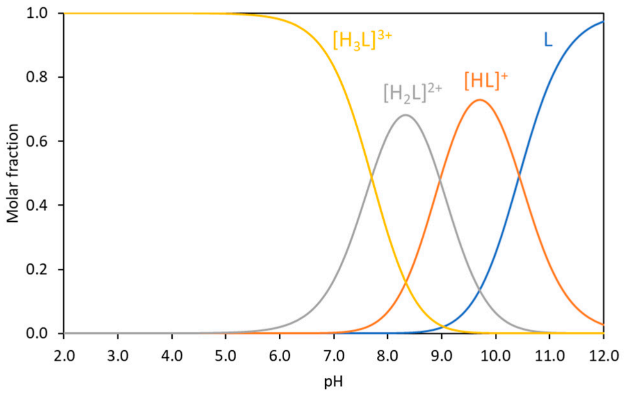

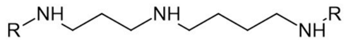

1a

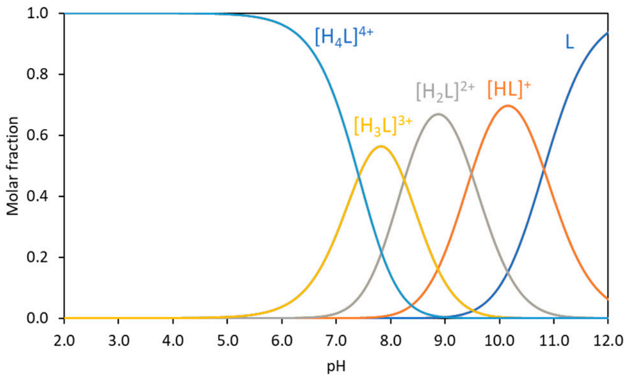

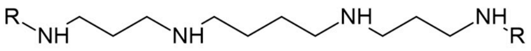

2a

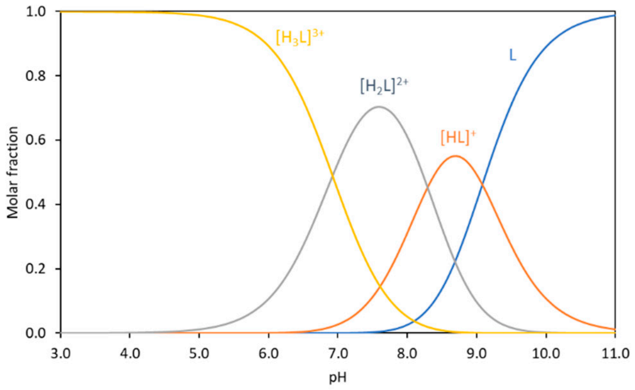

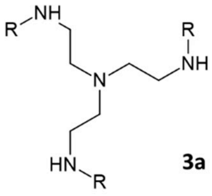

3a

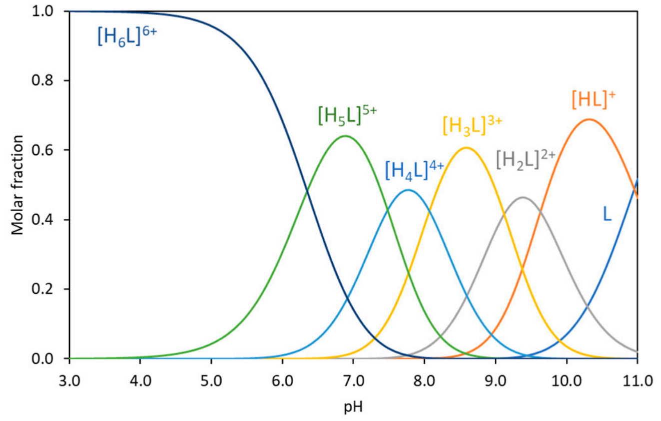

[H$_6$L]$^{6+}$
[H$_5$L]$^{5+}$
[H$_4$L]$^{4+}$
[H$_3$L]$^{3+}$
[H$_2$L]$^{2+}$
[HL]$^+$
L
Molar fraction
pH

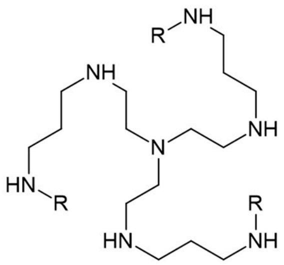

NH
R
HN
N

4a

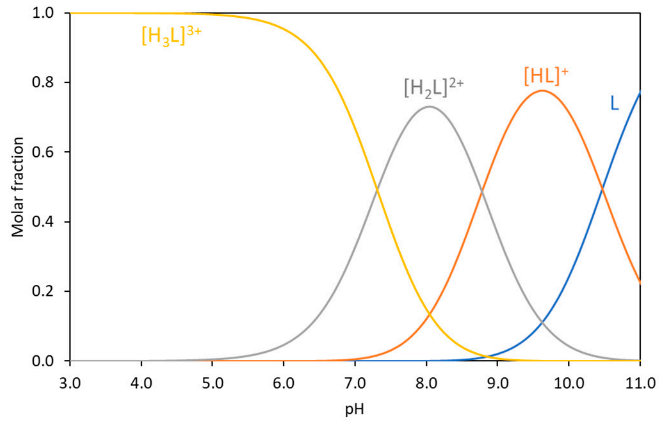

[H$_3$L]$^{3+}$
[H$_2$L]$^{2+}$
[HL]$^+$
L
Molar fraction
pH

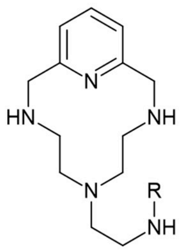

N
HN
NH
R

5a

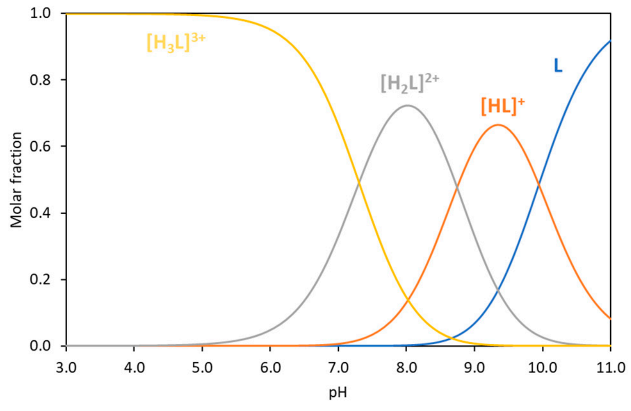

[H$_3$L]$^{3+}$
[H$_2$L]$^{2+}$
[HL]$^+$
L
Molar fraction
pH

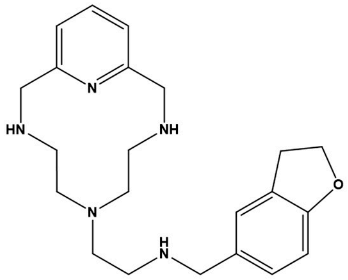

N
HN
NH
O

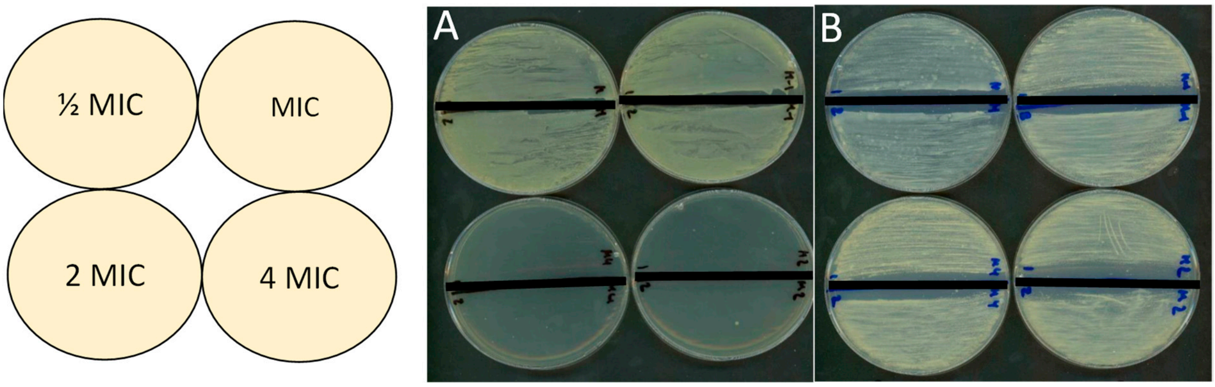

Figure S6. Determination of the minimal microbicidal concentration. On the left, a scheme of the plates used for these experiments is shown. In each sector 30 µL of a 10-fold dilution of each one of the tubes described in the experimental section were applied. On the right, representative images of the resulting growth in plate are shown. (A) Compound **4a** in *S. aureus*. (B) Compound **4a** in BY4741.

A

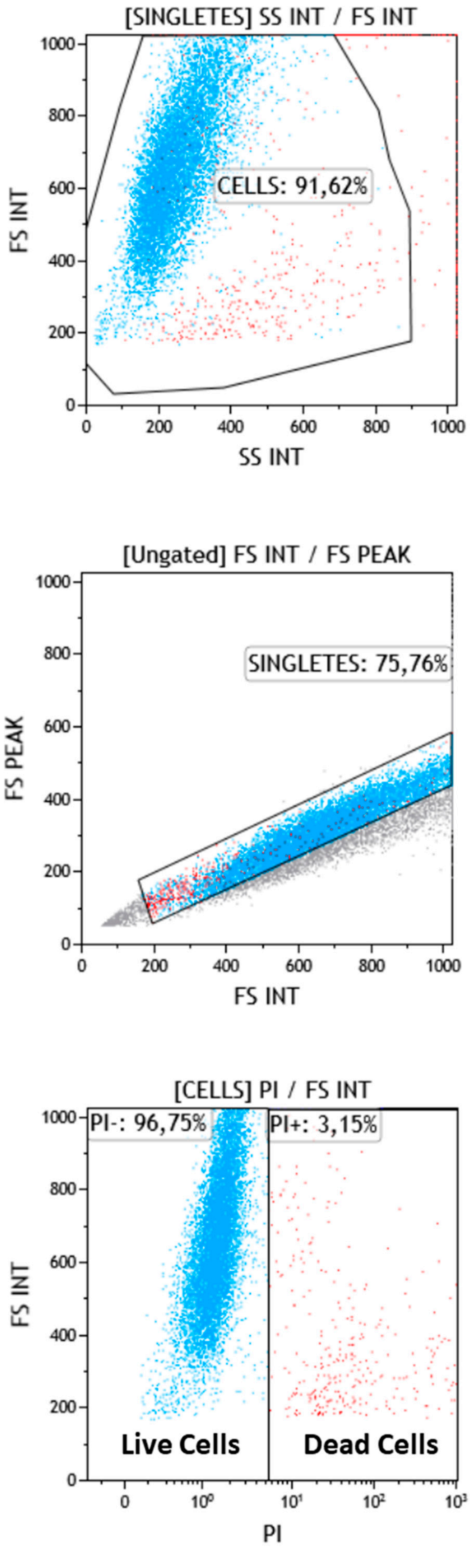
[SINGLETES] SS INT / FS INT
CELLS: 91,62%
FS INT
SS INT
[Ungated] FS INT / FS PEAK
SINGLETES: 75,76%
FS PEAK
FS INT
[CELLS] PI / FS INT
PI-: 96,75%
PI+: 3,15%
Live Cells
Dead Cells
FS INT
PI

B

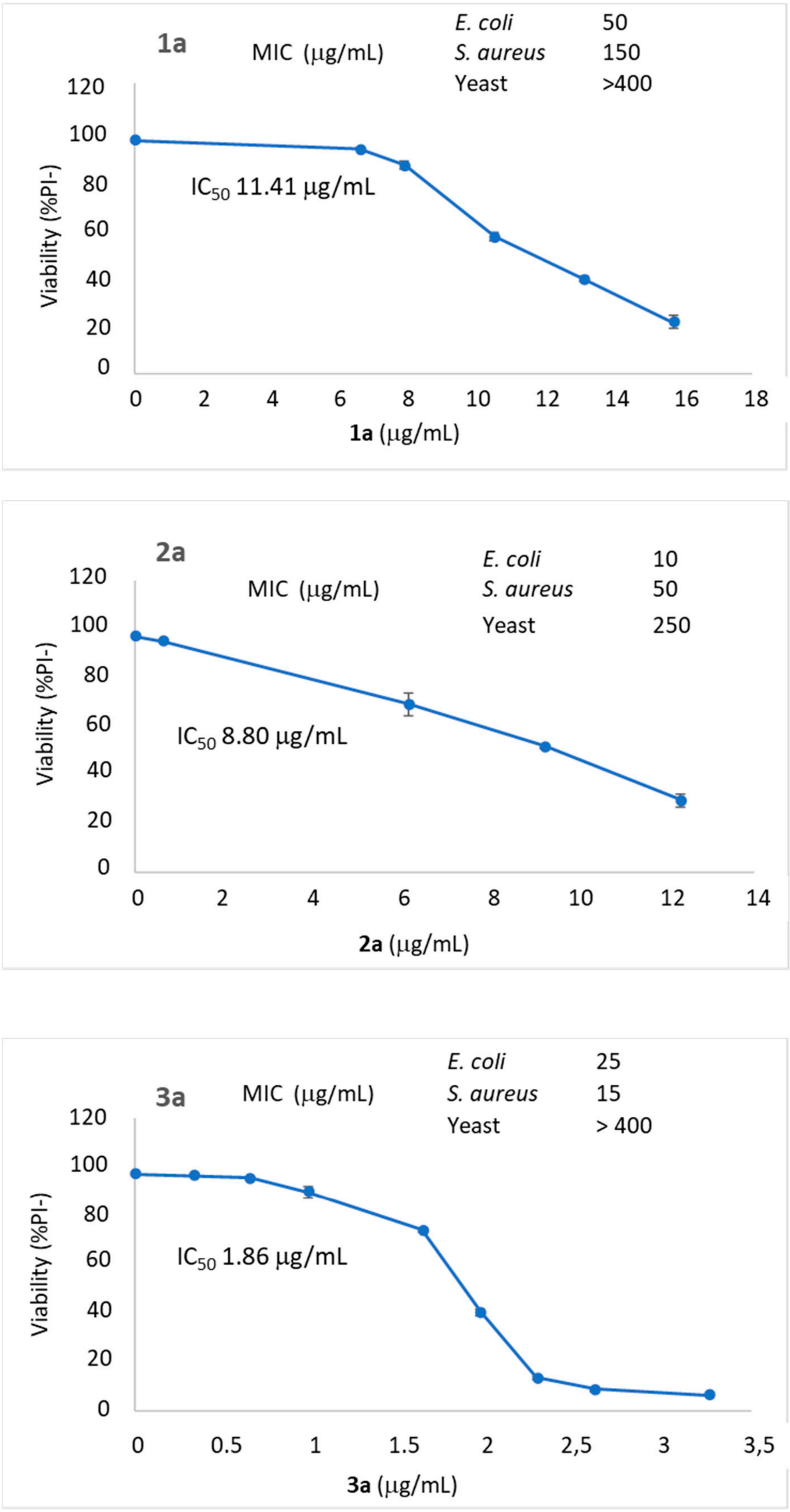
1a
MIC (µg/mL)
E. coli 50
S. aureus 150
Yeast >400
IC50 11.41 µg/mL
Viability (%PI-)
1a (µg/mL)
2a
MIC (µg/mL)
E. coli 10
S. aureus 50
Yeast 250
IC50 8.80 µg/mL
Viability (%PI-)
2a (µg/mL)
3a
MIC (µg/mL)
E. coli 25
S. aureus 15
Yeast > 400
IC50 1.86 µg/mL
Viability (%PI-)
3a (µg/mL)

Bcont

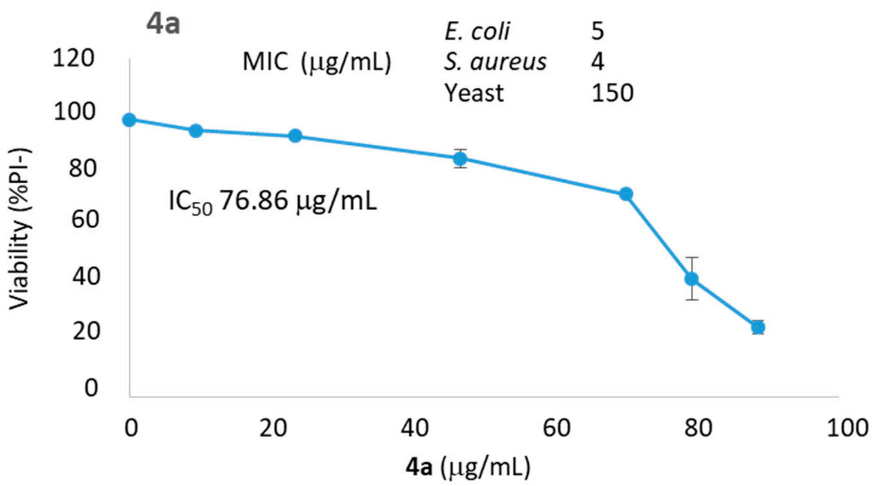

4a
MIC (µg/mL)
E. coli 5
S. aureus 4
Yeast 150
IC50 76.86 µg/mL
Viability (%PI-)
4a (µg/mL)

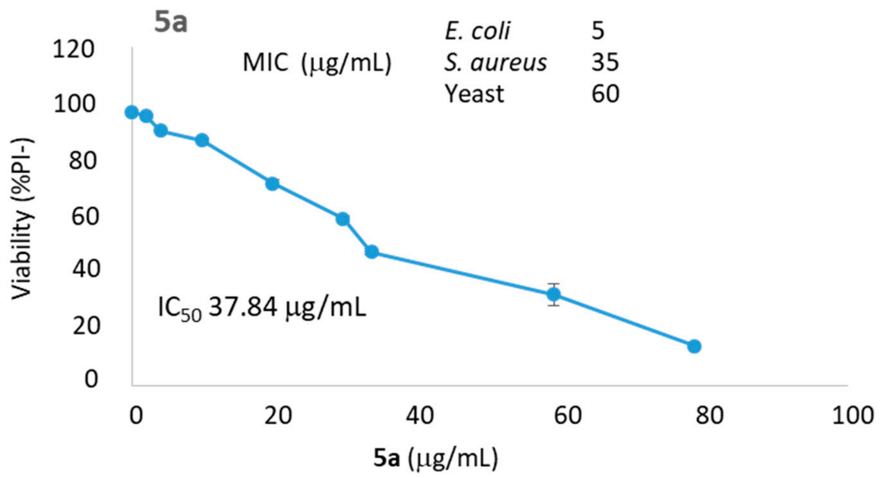

5a
MIC (µg/mL)
E. coli 5
S. aureus 35
Yeast 60
IC50 37.84 µg/mL
Viability (%PI-)
5a (µg/mL)

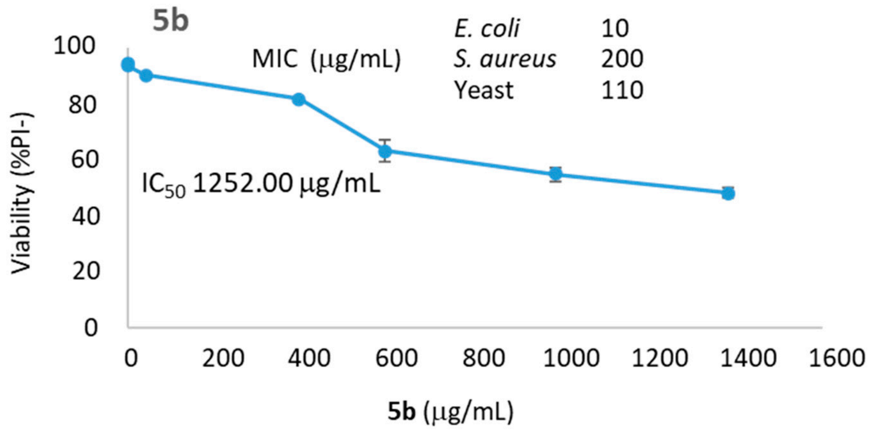

5b
MIC (µg/mL)
E. coli 10
S. aureus 200
Yeast 110
IC50 1252.00 µg/mL
Viability (%PI-)
5b (µg/mL)

Bcont

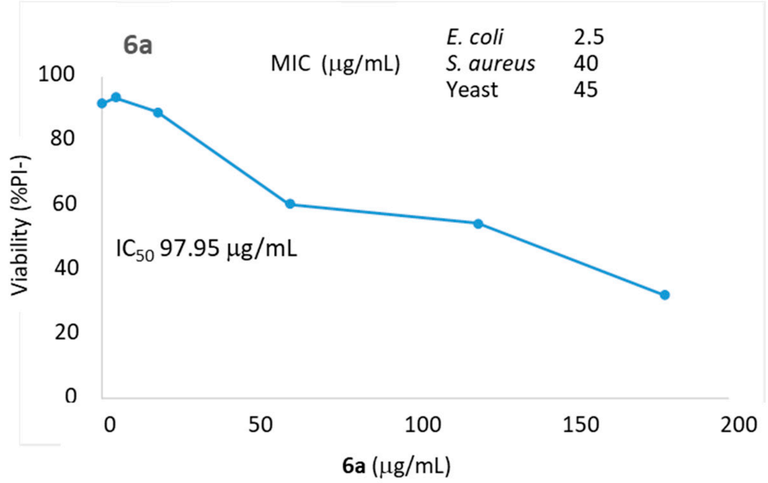

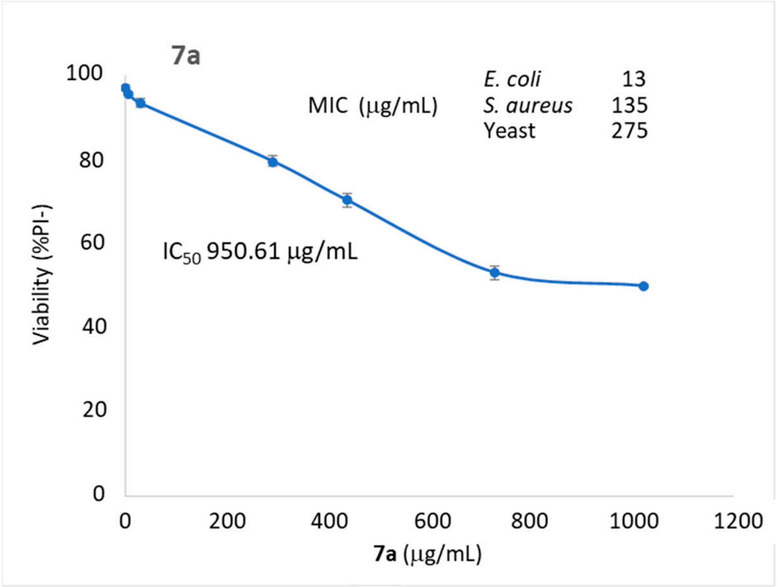

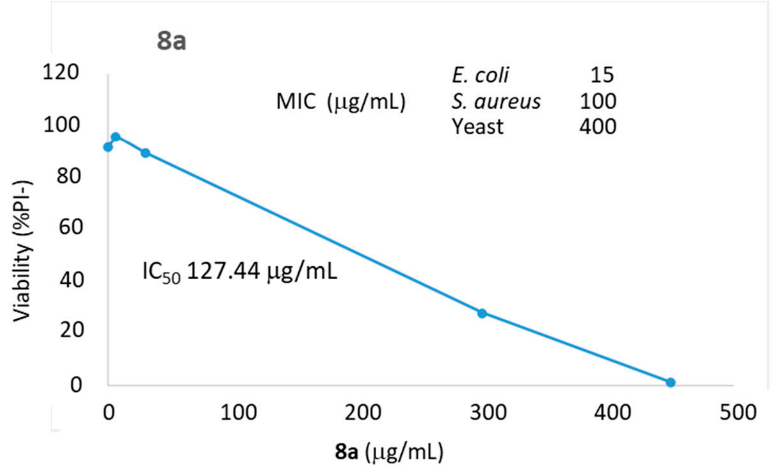

Figure S7. Cytotoxicity determination by FACS. (A) Image of the information provided by these analyses. (B) Graphs showing the variation of the Jurkat cells viability in function of the concentration of the compounds tested.
